# Detecting medication errors associated with the use of beta-lactams in the Russian Pharmacovigilance database

**DOI:** 10.1186/s40360-020-00470-x

**Published:** 2021-01-08

**Authors:** Anna V. Kuzmina, Irina L. Asetskaya, Sergey K. Zyryanov, Vitaliy A. Polivanov

**Affiliations:** 1 Pharmacovigilance Center, Information and Methodological Center for Expert Evaluation, Record and Analysis of Circulation of Medical Products under the Federal Service for Surveillance in Healthcare, 4-1 Slavyanskaya Square, Moscow, Russian Federation 109074; 2 Department of General and Clinical Pharmacology, Peoples’ Friendship University of Russia (RUDN University), 6 Miklukho-Maklaya Street, Moscow, Russian Federation 117198

**Keywords:** Spontaneous reports, Medication errors, Beta-lactams, Antibiotics, Russia

## Abstract

**Background:**

Comprehensive analysis of all available data in spontaneous reports (SRs) can reveal previously unidentified medication errors (MEs).

**Methods:**

To detect MEs, we performed a retrospective analysis of SRs submitted to the Russian pharmacovigilance database in the period from January 01, 2012, to August 01, 2014. This study evaluated SRs of cases where beta-lactam antibiotics were the suspected drug.

**Results:**

A total of 3608 SRs were analyzed. MEswere detected in 1043 reports (28.9% of all cases). The total number of detected errors was 1214. Reporters themselves indicated MEs in 29 SRs. A term denoting an ME was selected in the “Adverse Reactions” section in 18 of these SRs, whereas in the other 11 reports information on the ME was found only in the “Case narrative” section.

MEs were associated with wrong indications in 32.5% of the cases; 61.0% of these cases were viral infections. Various dosing regimen violations constituted 29.7% of MEs. A contraindicated drug was administered in 17.3% of all detected MEs, most commonly to a patient with a history of allergy to the suspected drug or severe hypersensitivity reactions to other drugs of the same group.

**Conclusion:**

Automatic identification of MEs in the pharmacovigilance database is sometimes precluded by the absence of a code for the respective episode in the “Adverse Reactions” section, even when the error was detected by the reporter. The most frequent types of MEs associated with the use of beta-lactams in Russia are the leading risk factors of growing bacterial resistance.

## Background

According to the European Medicines Agency, medication errors (MEs) constitute the main preventable cause of adverse drug reactions (ADRs) [1]. Evaluation of the most common errors permits identification of top-priority problems in this field and development of measures to decrease the occurrence of MEs and minimize risks associated with inappropriate use of medicinal products. Errors in the use of antibacterial drugs deserve special attention. Inappropriate use of antibiotics may have unfavorable consequences not only for the patient (development of ADRs, generalization of infection in the event of treatment failure) but for health care as a whole, because this results in growing microbial drug resistance. Safety issues related to MEs are usually detected in the post-marketing period, and spontaneous reports (SRs) are an essential source of data during this time, as they provide information on the use of the drug in real-life clinical practice. Many countries have clear requirements for reporting suspected serious and non-serious ADRs resulted from MEs to pharmacovigilance centres.

The Federal Service for Surveillance in Healthcare (Roszdravnadzor) carries out monitoring of medicines’ safety and effectiveness in the Russian Federation. Pharmacovigilance Center under Roszdravnadzor evaluates and analyzes information obtained through safety monitoring, including spontaneous reports of adverse reactions to medicines, vaccines, herbal products submitted to the Russian pharmacovigilance database (Automatic Information System “Pharmacovigilance”) by healthcare professionals, pharmaceutical companies and patients. The results of monitoring are sent to the Ministry of Health (Minzdrav) of Russia to take decisions and regulatory actions A requirement to report ADRs to Roszdravnadzor, including MEs, is laid down in the Federal Law No. 61-FZ “On Circulation of Medicines” [2] and in the Eurasian Economic Union “Good pharmacovigilance practice” guideline [3] Generally it is the same requirement as in the European Union [4]. The national pharmacovigilance database has been in operation since 2008, and it should be mentioned that reporting of ADRs to this system has significantly increased in recent years. In 2020 our database holds more than 230,000 reports. It has been shown that ADRs in a number of SRs are related to MEs. These errors are not always apparent, and therefore they are not recognized by the reporters [5].

At the first stage, we conducted a retrospective analysis of SRs with ADRs to beta-lactams, as this is one of the most commonly used groups of antibiotics in medical practice. The objective of the study was to identify medication errors associated with the use of beta-lactams in the Russian pharmacovigilance database, and to describe the characteristics of these events.

## Methods

### Definitions

For this study, we used the following definitions:

*Medication error* is an unintended failure in the drug treatment process that leads to, or has the potential to lead to, harm to the patient [4, 6].

*Adverse reaction* is a response to a medicinal product, which is noxious and unintended. Adverse reactions may arise from use of the product within or outside the terms of the marketing authorization or from occupational exposure. Use outside the marketing authorization includes off-label use, overdose, misuse, abuse and medication errors [4].

*Causality:* In accordance with ICH-E2A, the definition of an adverse reaction implies at least a reasonable possibility of a causal relationship between a suspected medicinal product and an adverse event1. An adverse reaction, in contrast to an adverse event, is characterised by the fact that a causal relationship between a medicinal product and an occurrence is suspected. For regulatory reporting purposes, as detailed in ICH-E2D, if an event is spontaneously reported, even if the relationship is unknown or unstated, it meets the definition of an adverse reaction. Therefore all spontaneous reports notified by healthcare professionals or consumers are considered suspected adverse reactions, since they convey the suspicions of the primary sources, unless the reporters specifically state that they believe the events to be unrelated or that a causal relationship can be excluded [4].

### Study design

This is a retrospective descriptive study.

### Data selection

We evaluated SRs submitted to the Russian pharmacovigilance database in the period from January 01, 2012, to August 01, 2014.

The inclusion criterion for SRs in this study was the presence of one or several beta-lactam antibiotics registered in the Russian Federation among the drugs suspected to have caused an ADR. The database was searched for the following international nonproprietary names (INN): benzylpenicillin, benzathine benzylpenicillin, phenoxymethylpenicillin, oxacillin, azlocillin, ampicillin, ampicillin/oxacillin, amoxicillin, ampicillin/sulbactam, amoxicillin/clavulanate, amoxicillin/sulbactam, piperacillin/tazobactam, ticarcillin/clavulanic acid, ceftriaxone, cefotaxime, cefazolin, cefoperazone/sulbactam, cefuroxime, ceftazidime, cefepime, cefoperazone, cefixime, ceftibutene, cefalexin, cefamandol, cefpyrom, ceftarolinefosamil, cefoxytin, cefaclor, cefditoren, cefpodoxime, ceftizoxime, ceftobiprol, meropenem, imipenem/cilastatin, ertapenem, doripenem, aztreonam.

Duplicate and invalid SRs were excluded from the study. Validity was determined according to paragraph VI.B.2 of the “Guideline on good pharmacovigilance practices” and paragraph 7.1.2 of the Eurasian Economic Union “Good pharmacovigilance practice”, which state that information in a SR must contain at least 4 elements: identifiable reporter; identifiable patient; at least one suspected drug; at least one suspected ADR. If any of these 4 elements is absent, the report is considered invalid [3, 4]. It should be noted that the identifiers of the patient were initials (not full name), date of birth, age or gender. Suspect drugs were identified by brand name and INN, which are both selected automatically by reporters when they fill out the official reporting Form. For this study, we used INN of beta-lactam antibiotics. We also excluded cases of ADRs that occurred in any clinical studies (both before and after approval) and in foreign countries.

### Identification and types of medication errors

During this study, experts of the Roszdravnadzor performed a complete analysis of information in all sections of the selected SRs to find medication error data related to the use of beta-lactam antibiotics. MEs associated with the use of an antibacterial drug were detected based on the summary of product characteristics (SmPC) approved in the Russian Federation, as well as care standards and clinical guidelines for particular diseases that were encountered in this study.

We divided all detected errors into the following types: administration of an antibiotic in the absence of indications/wrong indication; administration of a contraindicated drug; incorrect dosage and regimen; late discontinuation of a drug in a patient developing an ADR; late or irrational switch of antibiotic in the event of treatment failure; incorrect assessment of treatment efficacy; irrational switch of an antibacterial drug; incorrect preparation of an antibiotic solution; administration of a drug through a route not specified in the SmPC; irrational combination of drugs; administration of a treatment regimen unsuitable for the disease/improper treatment strategy; incorrect choice of dosage form for an antibiotic; accidental, unintended drug use; violation of the storage conditions for an antibiotic.

It should be mentioned that errors due to late discontinuation of a drug included cases where the antibiotic was continued despite manifestations of allergy or severe ADRs not classified as hypersensitivity reactions.

Rationality of a switch of antibiotics described in an SR was evaluated based on the clinical situation, information in the “Case narrative” section, and in some cases (when this information was available), bacteriological test results.

According to conventional recommendations, the first efficacy assessment is performed 48–72 h after the start of antibacterial therapy. The main clinical efficacy criteria are reduction of disease symptoms, body temperature lowering, and improvement in laboratory test results indicative of the severity of inflammation. If no improvement is observed within the first 3 days of antibiotic therapy, the treatment plan should be revised [7, 8]. Errors due to late switch of an antibiotic included cases where the patient continued taking the drug for more than 3 days in the absence of any signs of efficacy of the antimicrobial therapy.

Some errors arised from incorrect assessment of treatment efficacy. These errors included the cases when reporters indicated inappropriate parameters/indicators as efficacy criteria. The example of such error is no improvement in gingival oedema that followed tooth extraction or persistent cough in bronchitis.

In those cases when an ADR required early withdrawal of the medication, duration of antibiotic therapy was not evaluated. If an ADR developed on the first day of treatment and resulted in withdrawal of a suspected drug, dosing frequency and daily dose of the antibiotic were not calculated.

Assessments were carried out by three independent experts in all unclear situations.

### Statistical analysis

Descriptive statistics were used for all analyzed parameters; qualitative variables were described using absolute (n) and relative (%) values. Since reports are collected spontaneously, it is impossible to estimate the size of the general population.

## Results

A total of 37,500 SRs were submitted to the Russian pharmacovigilance database in the study period from January 01, 2012, to August 01, 2014.

The study included 3608 SRs with ADRs to beta-lactams (9.6% of all SRs obtained during the study period). The suspected drug was a penicillin derivative in 1123 cases, a cephalosporin in 2324 cases, and a carbapenem in 161 cases; there were no SRs to antibiotics of the monobactam subclass (only one such agent, aztreonam, has been registered in Russia).

Experts analyzing SRs with ADRs to beta-lactam antibiotics detected MEs in 1043 reports, i.e., 28.9% of the cases. It is important to underline that reporters themselves indicated errors associated with drug therapy only in 29 SRs (2.8% of SRs with detected MEs, 0.8% of all beta-lactam-related SRs). A term denoting an ME was selected in the “Adverse Reactions” section in 18 SRs (0.5% of all SRs included in the study), whereas in the other 11 reports (0.3% of all SRs included in the study) information on the ME was found only in the “Case narrative” section of the SR. Most SRs described 1 ME, 15.1% of the reports provided information about two or more simultaneous errors: 144 SRs (13.8%) about 2 MEs, 12 SRs (1,2%) about 3 MEs, and 1 SR (< 0,1%) about 4 MEs. Therefore, the total number of detected errors was 1214.

**Fig. 1:**
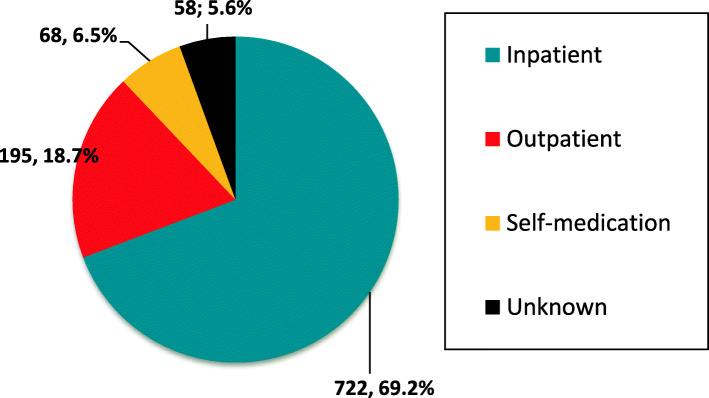
Distribution of spontaneous reports with medication errors by care stage

The distribution of SRs describing MEs by care stage is presented in Fig. 1.

SRs with detected MEs described female patients in 59.5% of the cases and males in 38.4%; no gender information was available in 2.1% of the reports. Similar ratios were observed in all subgroups of beta-lactams.

Most errors were detected in reports describing children under 18 years of age who were administered antibiotics (43.8% of the cases, 457 SRs). Patients aged 18–64 years were described in 42.2% of SRs with MEs (440 SRs). The proportion of SRs with MEs in patients aged ≥ 65 years was 10.7% (112 SRs). The age of patients was not indicated in 3.3% of the cases (34 SRs).

The distribution of detected MEs by type is presented in Table 1.

**Table 1:** Types of medication errors associated with the use of beta-lactam antibiotics

Type of medication error	Number of cases(n)	%
Administration of an antibiotic in the absence of indications/wrong indication	395	32.5%
Incorrect dosage and regimen	360	29.7%
Administration of a contraindicated drug	210	17.3%
Late discontinuation of a drug in a patient developing an ADR	80	6.6%
Irrational switch of an antibiotic	59	4.9%
Late or irrational switch of antibiotic in the event of treatment failure	31	2.6%
Incorrect preparation of an antibiotic solution	27	2.2%
Incorrect assessment of treatment efficacy	13	1.1%
Administration of a treatment regimen unsuitable for the disease/ Improper treatment strategy	11	0.9%
Irrational combination of drugs	10	0.8%
Administration of a drug through a route not specified in the SmPC	8	0.7%
Accidental, unintended drug use	5	0.4%
Violation of the storage conditions for an antibiotic	4	0.3%
Incorrect choice of dosage form for an antibiotic	1	0.1%
Total	1214	100%

*ADR* adverse drug reaction, *SmPC* summary of product characteristics

Thirty two point five percent of all detected MEs were associated with wrong indication for use of antibacterial drug (Table 1). Antibiotics were used to treat viral infections in 60.5% of these cases (*n*=239): 53.7% of the cases (*n*=212) were acute respiratory viral infections, 2.8% (*n*=11) were other viral infections (unspecified), 2.8% (n=11) adenovirus infection, 1.0% (*n*=4) infectious mononucleosis, and 0.3% (n=1) influenza. Another 0.5% cases of this group (*n*=2) described use of an antibiotic to prevent an acute respiratory viral infection. Therefore, the total proportion of errors arising from use of an antibiotic in viral infections was 61.0% in this ME group (*n*=241). We also considered erroneous the practice of administering antibiotics for indications specified as fever (6.8%; *n*=27), inflammation (3.8%; *n*=15), cough (3.0%; *n*=12), sore throat (1.5%, *n*=6).

Incorrect dosage and regimen of antibiotics constituted a total of 29.7% of MEs detected in this study (*n*=360). Wrong dosing frequency accounted for 12.6% of all MEs (*n*=153); 12.4% of the MEs (*n*=151) were in patients given antibiotics at a lower dosing frequency than the one required by the SmPC. In 11.4% of the cases (*n*=138), patients received an antibiotic at a dose exceeding the recommended one. In another 3.9% of the cases (*n*=47), underdosage took place. There are objective difficulties involved in detecting MEs related to improper treatment duration in SRs, because the suspected drug is usually discontinued when an ADR develops: either treatment is withdrawn altogether or the drug is replaced with another. We were able to detect MEs of this kind only in reports (1.8% MEs; *n*=22) where ADRs were delayed, developing some time after the end of a full treatment course.

Contraindicated antibacterial drugs were administered in 17.3% of all detected ME cases (*n*=210), and 71.4% of these cases (*n*=150) were in patients with a history of allergy to the drug or severe hypersensitivity reactions to drugs of similar chemical structure.

In 6.6% of ME cases (*n*=80), the SRs contained information on late discontinuation of drug therapy in a patient developing an ADR that required withdrawal of the drug (mainly in the presence of manifestations of an allergic reaction to antibiotic). An irrational switch of antibiotic (disagreeing with the properties of the isolated pathogen or clinical guidelines for the respective disease) was detected in 4.9% of the cases (*n*=59). In 2.6% of the cases (*n*=31), errors were due to a late or irrational change of the drug in the presence of clear evidence of failure of the administered antibacterial therapy. An opposite situation was observed in 1.1% of the cases (*n*=13): a switch of antibiotic was unjustified as a result of incorrect assessment of the efficacy of treatment. Other types of MEs were significantly less frequent in the SRs, accounting for 3.2% of all ME cases in total.

## Discussion

One of the main disadvantages of the SR assessment method is a low percentage of reported ADRs, and this problem is particularly often in the case of ADRs caused by MEs [9]. In our study, a term denoting inappropriate use of a suspected drug was included in 0.5% of the SRs. Full expert analysis of all data included in the SRs revealed MEs in 28.9% of the SRs. These data indicate both an unwillingness of physicians and pharmacists to report MEs and the fact that MEs remain unnoticed by health care professionals in many cases. In a number of cases (37.9%), we observed incorrect coding of ADR data in the database, as medication error-related information was only found in the “Case narrative” section of the SR.

Our results revealed that the proportion of SRs describing erroneous use of beta-lactams at the inpatient care stage was higher than that for the outpatient care stage. From our point of view, this was due to the fact that the most common suspected drugs were injectable beta-lactam antibiotics intended for use in the treatment of severe and complicated infections in hospital settings. The proportion of MEs due to self-medication was 6.5%. It has been reported that in many countries antibiotics are sold without physician’s prescription in over 50% of all cases [10–14]. A number of previous studies also demonstrated errors arising from self-administration of antibiotics, such as use of an antimicrobial drug at an improper dose, unjustifiably short duration of treatment, and discontinuation of the drug upon the improvement of disease symptoms [15, 16]. Over-the-counter availability of antibacterial medicines and self-medication with antibiotics is a pressing problem in Russia, too. Although our study yielded a relatively low percentage of errors due to self-medication, the obtained results should be interpreted with caution, as Russian patients very rarely send spontaneous reports themselves. As a result, self-medication errors become known only when the patient develops a serious ADR requiring medical assistance.

Most patients in SRs with detected MEs were female. Our results agree with the data of a similar Moroccan study [9]. Besides, some literature sources indicate that women are more likely to develop ADRs than men [17–19].

Our study discovered a significant proportion of SRs describing MEs in children treated with beta-lactam antibiotics. This finding may be explained by the frequent use of this drug class in pediatrics. It should also be taken into consideration that the risk of MEs is higher in children than in adult patients, and this is due to a whole range of factors: many medicinal products are not available in special pediatric dosage forms; doses of most drugs have to be calculated based on the child’s body weight or body surface area; information on the use of many drugs in pediatric populations is limited [20–22]. Besides, some authors believe that excessively aggressive antibiotic therapy, i.e., unjustified administration of antibacterial drugs beginning from the very first days of an infant’s life, particularly in diarrhea and upper respiratory tract infections most of which are caused by viruses, is among the problems of pediatrics [23–25].

According to the results of this study, 32.5% of all detected MEs (*n*=395) were a result of the use of antibacterial agents in the absence of indications. In 61.0% of the cases (*n*=241), these drugs were administered in viral diseases, most commonly in acute respiratory viral infections. It has already been shown that preventive use of antibiotics in viral infections does not preclude bacterial complications, but can often lead to acquired antimicrobial resistance. It should also be remembered that the use of antibiotics in such cases, when the efficacy of treatment is extremely dubious and the risk of ADRs (including serious ones) remains, is associated with an unfavorable benefit - risk profile [26].

Medication errors associated with incorrect dosage and regimen of beta-lactam antibiotics were the second most common type of mistakes in the SRs (29.7% MEs; *n*=360); the most common subtype of ME in this group is inappropriate dosing frequency. It should be underlined that the special pharmacodynamic properties of all beta-lactams place them among agents with time-dependent antimicrobial activity. The basic parameter that determines the clinical and microbiological efficacy of these drugs is the time during which the blood concentration of the antibiotic exceeds its minimum inhibitory concentration for the respective pathogen [27, 28]. Therefore, it is essential to adhere to the recommended interval between doses rather than increase single doses in order to improve the antimicrobial efficacy of drugs of this class. Although failure of antibacterial therapy was observed only in 8 SRs where a decreased dosing frequency was shown, it should be remembered that such deviations are considered serious in international practice due to increased risk of development of resistant microbial strains [29]. We observed only one case where administration of an antibiotic at a lower-than approved dose had probably resulted in treatment failure, but underdosage of antimicrobials is also facing criticism within the discussion of the bacterial resistance issue.

Our results show that the quality of allergic history collection is still a pressing problem. What makes underestimation of previous drug therapy-related experiences so dangerous is that even a low single dose of a drug, particularly administered to a patient already sensitized to it, can trigger severe, and sometimes life-threatening, ADRs (anaphylactic shock, Lyell’s syndrome, Stevens - Johnson syndrome, etc.). Therefore, this type of errors should be considered serious as well. It is important to bear in mind that all antibiotics, and in particular beta-lactams, are among drugs associated with a high risk of allergic reaction.

MEs related to inappropriate timing of drug discontinuation following development of a number of complications of drug therapy (in particular severe, serious ADRs, allergies) may produce aggravation of ADR symptoms and require additional time and money to relieve the developing disorders.

An inappropriately timed change of antibiotic, in particular continuation of an ineffective antibacterial therapy, entails numerous negative consequences. Delay in administering a different, more suitable antibiotic results in progression of inflammation and development of complications, as well as longer time to recovery. In addition, there is an increased risk of adverse effects (toxicity), as well as of the development or worsening of antibiotic resistance. Continuation of therapy in spite of the absence of a noticeable improvement undermines patients’ and their relatives’ trust in the attending physician. It is also evident that such erroneous antibacterial therapy has poor cost-effectiveness (unnecessary spending of the ineffective drug, additional cost of treatment of toxic effects, etc.). At the same time, an unjustified switch of antibacterial therapy as a result of incorrect treatment efficacy assessment is also dangerous, especially in the context of the risk of developing microbial resistance.

We detected just a few errors related to administration of irrational drug combinations. Beta-lactam antibiotics are known to rather rarely interact with other drugs; there are no absolute incompatibilities involving representatives of this pharmacological class. Besides, in the current situation where we have a substantial armamentarium of highly effective broad-spectrum antibacterial drugs, the scope of indications for combination antibacterial therapy has significantly narrowed and monotherapy is still preferred in the treatment of many infections [30]. Therefore, the total number of SRs containing information on the use of drug combinations was small.

Spontaneous reporting is not always a suitable method to detect certain types of MEs, such as inappropriate switch of antibiotic therapy or incorrect treatment strategy. Most reports do not provide enough information to allow adequate assessment of the clinical situation as a whole or conclusions as to whether drug therapy was used correctly in each individual case.

## Conclusions

Although the reporting rate for problems caused by incorrect use of drug therapy was low, the pharmacovigilance database is still a valuable source of MEs information. Detailed analysis of all information contained in SRs leads to a significant increase in the ME detection rate, because in many cases errors remain unnoticed by the health care professionals. Automatic data retrieval is hampered by incorrect coding of MEs in the electronic system and recording of medication error-related information only in the “Case narrative” section. Therefore, pharmacovigilance education providers should focus on the need to include the term denoting the committed medication error apart from the term indicating the detected ADR when entering inappropriate drug therapy-related information into the “Adverse Reactions” section of the suspected ADR reporting form in the database.

This retrospective analysis of SRs demonstrated that the most common types of MEs associated with beta-lactam therapy were: administration of an antibiotic in the absence of indications (most frequently for viral infections); deviations from the dosing schedule; and administration of a drug to a patient with a history of allergy to the suspected drug or to other drugs of the same group. All these types of MEs are serious. Excessive, irrational use of antibiotics and failure to comply with the recommended dosing regimen result in growing microbial drug resistance. Neglect of the patient’s allergic history is associated with high risk of serious hypersensitivity reactions. The obtained data indicating the predominant types of MEs enable optimization of education and advanced training programmes for health care professionals, as they point to the systemic, most common errors associated with beta-lactam therapy.

## Data Availability

The datasets used and analyzed during the current study are available from the corresponding author on reasonable request.

## Abbreviations

ADR: Adverse drug reaction
ME: Medication error
SmPC: Summary of product characteristics
SR: Spontaneous report
